# The Impact of Additional Respiratory Dead Space Volume Mask During Warm-Up on Skin Blood Flow and Choice Reaction Time in Cyclists: A Randomized Crossover Trial

**DOI:** 10.3390/jcm15114301

**Published:** 2026-06-02

**Authors:** Rafał Hebisz, Natalia Danek, Paulina Hebisz

**Affiliations:** 1 Department of Sport Didactics, Faculty of Physical Education and Sport, Wroclaw University of Health and Sport Sciences, 51-612 Wroclaw, Poland; rafal.hebisz@awf.wroc.pl; 2 Department of Physiology and Biomechanics, Faculty of Physical Education and Sport, Wroclaw University of Health and Sport Sciences, 51-612 Wroclaw, Poland; paulina.hebisz@awf.wroc.pl

**Keywords:** physiological stress, blood redistribution, exercise thermoregulation

## Abstract

**Background**: This study aimed to assess skin blood flow (SkBF) and choice reaction time (RT) after breathing through an increased respiratory dead space volume, following warm-up and prior to intense exercise. **Methods**: A group of 24 cyclists completed two exercise tests on a cycle ergometer, each at a workload of 110% of their maximal power (110%Pmax) determined during a graded test. A 15 min warm-up and an 8 min passive recovery period preceded both tests. During the recovery period before one of the tests, participants breathed through an increased respiratory dead space volume (ARDSv) of 1000 mL, while no breathing modification (non-ARDSv) was used before the other test. During the tests, measurements included skin blood flow (SkBF), body surface temperature (T), heart rate variability (HRV) parameters, and choice reaction time (RT). In both experimental protocols, main and mixed effects were detected across five repeated SkBF measurements (taken during the warm-up, the first half of recovery, the second half of recovery, during the 110%Pmax test, and in post-test recovery). **Results**: The analysis revealed higher HR and lower SDNN values (*p* < 0.05) during the post-warm-up rest period in the ARDSv protocol compared to the non-ARDSv protocol. The Friedman analysis of variance showed statistically significant effects of repeated measurements of SkBF in the non-ARDSv test (χ^2^ = 52.37; df = 4; *p* = 0.00; W = 0.55) and in the ARDSv test (χ^2^ = 64.1; df = 4; *p* = 0.00; W = 0.67). Similar effects were obtained in the T analysis. Post hoc tests showed that SkBF and T at restitution after the 110% Pmax test were statistically significantly higher than SkBF and T during the 110% Pmax test only in the ARDSv protocol. Analysis of variance revealed a repeated-measures effect for mean RT (ƞ^2^ = 0.21; df = 1; *p* = 0.00; F = 11.97) and covariance analysis showed that baseline mean RT was a strong predictor of outcome mean RT, while the study protocol was a weak predictor of post-exercise mean RT. **Conclusions**: Higher HR and lower SDNN during the period between warm-up and the 3 min test suggest increased physiological strain associated with the ARDSv procedure. Furthermore, only weak and inconclusive effects were observed for skin blood flow and choice reaction time responses following ARDSv application.

## 1. Introduction

Previous scientific studies indicate that breathing through an additional respiratory dead space volume (ARDSv), increased by 1000 mL between warm-up and intense exercise, may modify the metabolic response of the body [1,2]. Such breathing allows intense exercise to be performed with higher oxygen uptake, a lower respiratory exchange ratio, a slower development of acid–base imbalance, and a reduced perception of effort compared with standard conditions (without ARDSv) [1,2]. These effects were reported at the group level. Individual case variation in response to ARDSv was observed [1,2]. As a potential mechanism underlying these effects, vasodilation in skeletal muscles in response to mild hypercapnia was initially suggested [1]. However, further analyses demonstrated higher blood oxygen saturation and smaller changes in plasma volume following exercise preceded by breathing through ARDSv. These findings indicated dilation of pulmonary blood vessels and a reduction in hydrostatic pressure in the muscles [2]. Vasodilation in muscles or lungs during intense physical activity may be associated with systemic redistribution of blood flow during intense exercise due to well-described vasomotor control mechanisms [3,4,5,6]. However, based on the currently available literature, it is difficult to determine whether the increase in exercise oxygen uptake and blood saturation following the addition of ARDSv breathing to the warm-up occurs with simultaneous changes in temporal skin blood flow. Previous studies have shown that temporal blood flow during intense exercise and recovery is related to power output in progressive tests and repeated sprinting efforts [7]. These results raise the question of whether temporal blood flow during intense exercise may be modulated by respiratory effects in ARDSv [7]. Existing studies only indicate that skin blood flow does not change under hypercapnic conditions at rest [8]. There is, however, a lack of data on exercise-induced changes in temporal skin blood flow in response to ARDSv breathing or hypercapnia induced by other means.

Breathing through ARDSv induces hypercapnic conditions [9]. Hypercapnia at rest [10] and during intense exercise [11] has been shown to increase cerebral blood flow. Changes in cerebral blood flow can affect cognitive functions, including reaction time to a stimulus [12,13]. Changes in reaction time may be particularly evident in tasks requiring complex information processing; therefore, exercise studies tend to yield larger statistical effects when using choice reaction time tests compared with simple reaction tests [13]. The literature does not provide information on whether reaction time changes after intense exercise preceded by ARDSv breathing or other air mixtures with increased CO_2_ partial pressure. Obtaining such data are important given the role of cognitive functions during physical activity, including in sport.

Potential changes in temporal skin blood flow and choice reaction time may be associated with athletes’ exercise capacity. Changes in temporal skin blood flow correlate with the power of maximal exercises [6], while reaction time may influence performance effectiveness, particularly under changing conditions [14]. Therefore, the present study aimed to evaluate temporal skin blood flow and choice reaction time following ARDSv use, after warm-up, and before intense exercise. It was hypothesized that ARDSv would modify temporal skin blood flow. Furthermore, it was assumed that ARDSv would cause changes in choice reaction time following intense exercise.

## 2. Materials and Methods

### 2.1. Participants

Before initiating this study, the minimum required sample size was calculated using G*Power 3.1.9.7 software. Because there were no previous studies examining the relationship between ARDSv and skin blood flow parameters, there were no empirical data to estimate the expected effect size a priori. Therefore, the calculation was based on conventional thresholds for large statistical effects (η^2^ ≈ 0.14, Cohen’s d ≈ 0.8). Assuming a statistical power of 80%, this study required at least 20 participants. Ultimately, 24 cyclists (aged 18–22 years) were recruited to account for dropouts or exclusions from the analysis. The mean body mass of participants was 69.2 ± 7.1 kg, mean height 178.2 ± 6.3 cm, and mean maximal oxygen uptake 58.9 ± 5.5 mL·kg^−1^·min^−1^. The athletes’ performance level was rated as 3 on a five-point scale proposed by De Pauw et al. [15]. Athletes were recruited because regularly trained individuals are less susceptible to familiarization and training adaptation effects [16] compared with novices [17].

Each participant could be excluded following an interview and analysis of blood pressure and hematological results. It was assumed that eligible participants must not have experienced infection within the 10 days preceding this study, must have a diastolic blood pressure below 90 mmHg, and a hematocrit below 50%. Additionally, cyclists were required to have experience in performing graded exercise tests on a cycle ergometer (at least two tests per year, including one within two months prior to participation).

This study began with 24 cyclists; all completed the tests and none withdrew during the study process.

All participants were informed about the study procedures and provided written informed consent. This study was conducted in accordance with the Declaration of Helsinki and received approval from the University Research Ethics Committee.

### 2.2. Experimental Procedure

**Figure 1:**
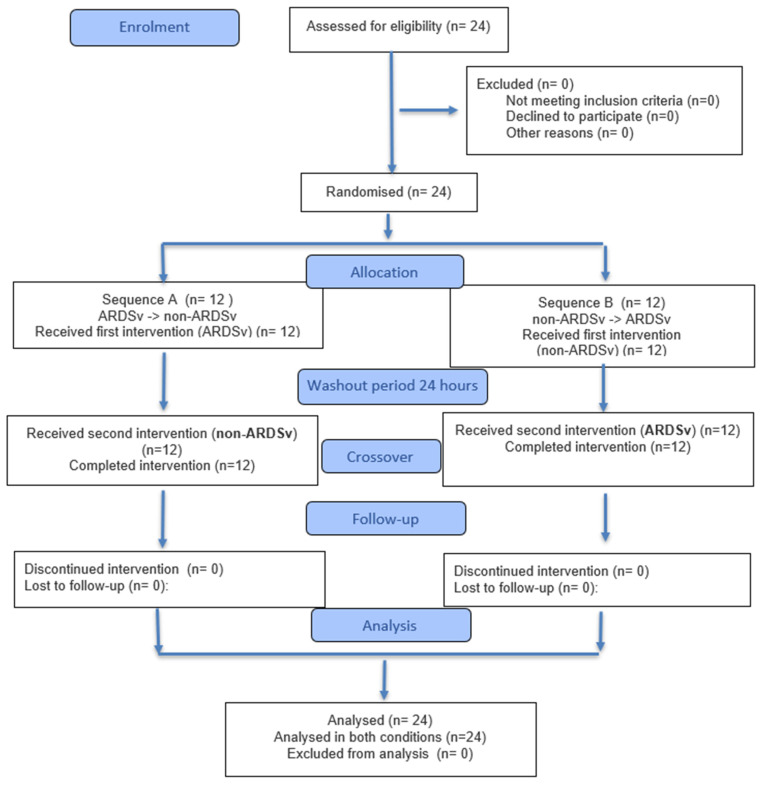
Participant flow diagram of the randomized crossover trial.

**Figure 2:**
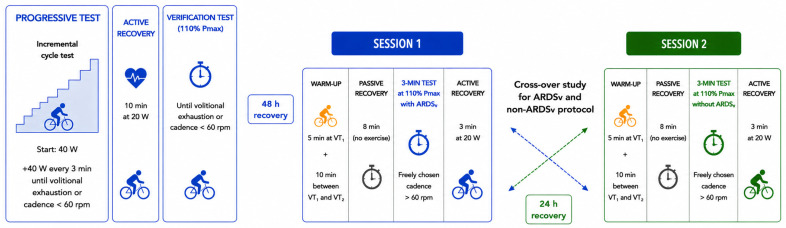
Diagram illustrating the study design.

During the first visit to the laboratory, preliminary examinations were conducted, including measurements of anthropometric and blood morphology parameters, blood pressure, and a medical interview. After completion of the preliminary examinations and qualification for participation in the main research procedure, a graded progressive test was performed. Following the progressive test, participants rested for 48 h. Subsequently, two constant-load tests lasting 3 min each were conducted at an intensity corresponding to 110% of the maximal power obtained in the progressive test (110%Pmax). A power output of 110%Pmax was chosen, which was 10% above the progressive test power, to ensure that participants would achieve maximal oxygen uptake in a short 3 min exercise test. Furthermore, during races, cyclists often achieve power levels close to 110% Pmax when riding uphill. One of the tests was preceded by breathing through an increased respiratory dead space volume (ARDSv test), whereas no breathing modifications were applied before the other test (non-ARDSv test). A 24 h interval was maintained between these tests. Participant flow through the randomized crossover study is presented in Figure 1. All randomized participants completed both intervention periods and were included in the final analyses. Before each 110%Pmax test, after a standardized warm-up, participants rated their perceived exertion (RPE) using the Borg 6–20 scale. For all cyclists, RPE values after the warm-up differed by no more than 1 point between tests. Additionally, before each test, participants declared no unusually high fatigue or signs of incomplete recovery. The order of the 110%Pmax tests was determined using a crossover design with intervention alternation (Figure 2). The ARDSv tests and non-ARDSv tests were randomized. The order of the ARDSv and non-ARDSv conditions was determined using a computer-generated random allocation sequence in a balanced crossover design (1:1 allocation ratio). Participants were enrolled and assigned to intervention sequences by the investigators conducting the study procedures. Due to the crossover laboratory design, no additional allocation concealment procedure was applied. Blinding of participants and investigators was not feasible because the ARDSv condition required the use of a visible breathing apparatus and direct investigator involvement.

All exercise tests were performed on the same cycle ergometer Lode Excalibur Sport (Lode BV, Groningen, The Netherlands). All participants were familiar with this cycle ergometer, as only cyclists experienced in progressive cycling tests were recruited.

All tests were conducted between 9:00 a.m. and 12:00 p.m. The order of participants was monitored; for example, the cyclist who performed the progressive test at 9:00 a.m. also performed the subsequent tests at 9:00 a.m.

Throughout the entire testing procedure, the cyclists did not use any ergogenic aids. They were instructed not to consume caffeine, energy drinks, or any additional supplements. Participants were instructed to maintain their habitual diet and hydration practices throughout this study. Each participant consumed a meal at least 2.5 h prior to each exercise test. However, detailed dietary intake and hydration status were not formally controlled or recorded.

#### 2.2.1. Research Procedures—Progressive Test

The progressive test was performed on a Lode Excalibur Sport cycle ergometer (Lode BV, Groningen, The Netherlands). The initial workload was set at 40 W. Subsequently, the workload was increased by 40 W every 3 min. The test continued until the participant was unable to continue or until the pedaling cadence dropped below 60 rpm. Participants were advised to maintain a cadence above 80 rpm. A drop to 60 rpm was a significant change, so the test was stopped immediately (3–5 s). Maximal power (Pmax) was calculated based on the power output achieved in the final stage of the test. For this purpose, a formula described in previous studies was used, in which Pmax was defined as the highest power achieved during the test minus 0.22 W for each second missing to complete the final stage [1,2]. After completion of the test, active recovery followed, consisting of continued cycling at 20 W. After 10 min of recovery, a verification bout was performed to confirm maximal oxygen uptake. During this effort, intensity was set at 110% Pmax. The effort continued until voluntary exhaustion or until cadence dropped below 60 rpm [1,18].

Throughout the entire testing procedure, respiratory parameters were recorded breath-by-breath using a Cosmed Quark ergospirometer (Cosmed, Rome, Italy). Recorded variables included minute ventilation (VE), oxygen uptake (VO_2_), carbon dioxide output (VCO_2_), end-tidal partial pressure of oxygen (PETO_2_), and end-tidal partial pressure of carbon dioxide (PETCO_2_). To determine maximal oxygen uptake (VO_2_max), data were averaged over 30 s intervals. VO_2_max was defined as the highest VO_2_ value recorded during either the progressive test or the verification phase. Using the procedure described by Pallares et al. [19], two independent evaluators identified the first ventilatory threshold (VT1) at the point where a nonlinear increase in PETO_2_ and VE/VO_2_ occurred without simultaneous changes in PETCO_2_ and VE/VCO_2_. The second ventilatory threshold (VT2) was identified at the point where a nonlinear decrease in PETCO_2_ and an increase in VE/VCO_2_ occurred. In cases of disagreement between evaluators, a third independent assessor made the final determination of VT1 and VT2.

#### 2.2.2. Three min ARDSv Test

The 3 min ARDSv test was conducted under controlled environmental conditions (air temperature 21 °C, humidity 45%). Before measurements began, participants remained under these conditions for 30 min to stabilize skin blood flow and body surface temperature, as described by Lohman et al. [20]. The test was preceded by a warm-up consisting of 5 min at VT1 intensity and 10 min at an intensity between VT1 and VT2, as detailed in previous publications [1,2]. Following the warm-up, an 8-min passive recovery period was implemented. Immediately after the warm-up, participants were fitted with a mask connected to a tube (diameter 3 cm), which increased the respiratory dead space volume by 1000 mL (ARDSv). The ARDSv apparatus was removed 50 s before the start of the 3 min test performed at 110%Pmax. Immediately before the test, a blood sample was collected to assess acid–base balance, and the results of this measurement were described in a previous publication [2]. During both the warm-up and the 3 min test, participants cycled on the Lode Excalibur Sport ergometer at a freely chosen cadence above 60 rpm. After the 3 min test, active recovery was performed, consisting of 3 min of cycling at 20 W.

Throughout the entire procedure, skin blood flow (SkBF) and body surface temperature (T) were measured using a Perimed Periflux 6000 laser Doppler flowmeter (Perimed AB, Järfälla, Stockholm, Sweden) equipped with a 6010 LDPM/Temp module. Data were recorded using a single Perimed 457 Small Angled Thermostatic Probe placed on the temple. The probe contained a Class I laser in accordance with EN(IEC) 60825-1:2014 [21]. The laser emitted an invisible, divergent beam of near-infrared radiation with a maximum power of 5 mW, which underwent a frequency shift upon reflection from moving erythrocytes. The wavelength of the emitted radiation was 785 nm. Laser Doppler signal analysis covered a frequency range of 5 Hz to 25 kHz. Due to the protocol’s duration, a sampling frequency of 32 Hz was used. SkBF data were expressed in arbitrary units (PUs). The probe was positioned 10 mm above and 30 mm lateral to the right lateral canthus. The selection of this site was based on previous analyses showing that changes in temple surface temperature accurately reflect changes in skin blood flow during short and dynamic exercise [7].

SkBF and T measurements were averaged for the following time intervals: 3 min preceding the warm-up (SkBF_b_ and T_b_); the last 3 min of the warm-up (SkBF_w-u_ and T_w-u_); the first half of the post-warm-up recovery period (SkBF_1_ and T_1_); the second half of the recovery period (SkBF_2_ and T_2_); the 3 min test at 110%Pmax (SkBF_3_ and T_3_); and 3 min of post-test recovery (SkBF_4_ and T_4_). Subsequently, changes in SkBF were calculated as follows: ΔSkBF0 = SkBF_w-u_ − SkBFb; ΔSkBF_1_ = SkBF_1_ − SkBF_w-u_; ΔSkBF_2_ = SkBF_2_ − SkBF_w-u_; ΔSkBF_3_ = SkBF_3_ − SkBF_2_; ΔSkBF_4_ = SkBF_3_ − SkBF_w-u_; ΔSkBF_5_ = SkBF_4_ − SkBF_3_. Analogous calculations were performed for T. The calculation of ΔSkBF was justified by statistical analyses indicating low repeatability of SkBF measurements and a high standard measurement error when measurements were compared across different days.

Simultaneously with SkBF recording, R-R intervals were recorded using a Polar V800 heart rate monitor (Polar Electro Oy, Kempele, Finland). Heart rate variability (HRV) parameters were calculated using Kubios HRV Standard 3.0.x software (Kubios Oy, Kuopio, Finland). A moderate artifact correction threshold was applied. Time-domain parameters included the standard deviation of NN intervals (SDNNs) and the root mean square of successive differences (RMSSDs). In addition, frequency-domain analysis was performed using the fast Fourier transform, including high-frequency power (HFP) and low-frequency power (LFP). These calculations were performed for the 3 min baseline phase before warm-up (SDNN_b_, RMSSD_b_, HFP_b_, LFP_b_) and for the recovery phase between warm-up and the 3 min 110%Pmax test (SDNN_break_, RMSSD_break_, HFP_break_, LFP_break_). Based on the recorded data, mean heart rate during baseline (HR_b_), mean heart rate during the recovery phase (HR_break_), peak heart rate (HR_peak_—mean of the last 10 s of the 110%Pmax test), and heart rate during the last 10 s of the first (HRR1), second (HRR2), and third (HRR3) minutes of recovery were calculated. The decrease in heart rate during the first minute of recovery was calculated as ΔHRR1 = HR_peak_ − HRR1. Analogous calculations were performed for ΔHRR2 and ΔHRR3 for the second and third minutes of recovery, respectively.

Choice reaction time (RT) was measured according to a procedure similar to that described by Hebisz et al. [22]. The test was conducted in a seated position on the cycle ergometer twice: 5 min before the warm-up and after 3 min of recorded recovery following the 110%Pmax test. Measurements were performed using the MCZR/ATB 1.0 m system (ATB INFO-ELEKTRO, Zabrze, Poland). The system generated three types of visual stimuli (green, orange, and red) and two types of auditory stimuli (high and low tones). Participants held response buttons in their hands. Stimuli were presented at a distance of 3 m. Participants were instructed to respond as quickly as possible to a red light by pressing the button in the right hand and to a green light by pressing the button in the left hand, while ignoring all other stimuli. The RT test was preceded by a familiarization procedure conducted on the day of the progressive test, using a different stimulus program from that used in the measurements analyzed. The actual RT assessment used a program that presented 40 stimuli in a fixed sequence over 120 s. The following variables were analyzed: baseline mean reaction time (avRT_b_), recovery mean reaction time (avRT_rec_), baseline fastest reaction time (fRT_b_), and recovery fastest reaction time (fRT_rec_).

#### 2.2.3. Three min Non-ARDSv Test

The 3 min non-ARDSv test was performed according to the same procedure as the 3 min ARDSv test. The only difference between the protocols was the absence of ARDSv during the recovery period between the warm-up and the 3 min 110%Pmax test.

### 2.3. Statistical Analysis

The repeatability of the measurements was assessed using the intraclass correlation coefficient *ICC(2.1)*—a two-way random-effects model evaluating the absolute agreement of single measurements. Data from the PeriFlux 6000 device, as well as RMSSD and SDNN (HRV) recorded before the first and second tests, were used for the calculations. Baseline data (recorded before the start of the warm-up) were analyzed separately. Data recorded during the last three minutes of the warm-ups were also analyzed separately. An *ICC(2.1)* value ≥0.75 was considered indicative of good repeatability [23]. The following formula was used for the calculations:

$$ICC\left(2.1\right)=\frac{MSB-MSE}{MSB+\left(nr-1\right)\cdot MSE+\frac{nr}{no}\cdot \left(MSR-MSE\right)}$$

where *MSB*—mean square between subjects, *MSE*—mean square error, *MSR*—mean square for repeated measures, *nr*—number of repeated measures, and *no*—number of participants. Subsequently, the standard error of measurement (*SEM*) and minimal detectable change (*MDC*) were calculated according to the formulas described by Furlan and Sterr [24]:

$$SEM={SD}_{pooled}\cdot \sqrt[{2}]{1-ICC(2.1)}$$

$${MDC}_{95}=SEM\cdot 1.96\cdot \sqrt[{2}]{2}$$

where *SD_pooled_* denotes the pooled standard deviation of two datasets (test and retest). Additionally, intra-individual (*CV_intra_*) and inter-individual (*CV_inter_*) coefficients of variation were calculated according to the guidelines described by Chow and Liu [25]:

$${CV}_{intra}=\frac{\sqrt[{2}]{\frac{\sum {SD}_{intra}^{2}}{n}}}{x}\cdot 100\%$$

$${CV}_{inter}=\frac{{SD}_{inter}}{x}\cdot 100\%$$

where *SD*—standard deviation, *n*—number of participants, and *x*—inter-individual mean value.

To indicate the differences between measurements in the non-ARDSv and ARDSv protocols, the formula for the absolute percentage difference between test and retest (*APD*) was used:

$$APD=\frac{test-retest}{(test+retest)/2}\cdot 100\%$$

Agreement between measurements from the standard non-ARDSv and ARDSv tests was also assessed using the Bland–Altman method [26]. For each participant, the mean of the two measurements and the difference between them (value from the ARDSv test − value from the non-ARDSv test) were calculated. The plot presents the differences relative to the mean values, along with the mean difference line (bias) and limits of agreement (LoA) corresponding to the interval ±1.96 × SD of the differences.

Further statistical analyses were performed using Statistica 13.1 software. The Shapiro–Wilk test was used to compare the distribution of the analyzed variables with a normal distribution. When the distribution deviated from normality, the Friedman analysis of variance for repeated measures and the Wilcoxon post hoc test with Bonferroni correction were applied. When adjusting *p*-values using the Bonferroni correction, the actual number of performed comparisons was used. Depending on the aim and hypotheses, five comparisons were applied (for example, SkBF_1_ and SkBF_w-u_, SkBF_2_ and SkBF_w-u_, SkBF_3_ and SkBF_w-u_, SkBF_3_ and SkBF_2_, and SkBF_4_ and SkBF_3_). When the distribution did not deviate from normality, repeated-measures ANOVA was used. When significant results were obtained, Bonferroni post hoc tests were performed. Repeated measures analyses were conducted separately for the non-ARDSv and ARDSv tests when repeatability indices (ICC2.1, CV, Bland–Altman, SEM) did not meet the adopted criteria, or a two-factor analysis was used when assumptions of repeatability and normality were met. Additionally, analysis of covariance (ANCOVA) was performed within the GLM model to assess whether the baseline values and the study protocol used were significant predictors of the outcome values the analyzed variables. Moreover, ANCOVA analyses were conducted for variables in which baseline values showed substantial associations with post-intervention outcomes and where baseline imbalance between experimental conditions could potentially influence interpretation of the intervention effect. When repeatability criteria were not satisfied, pairwise comparisons of results from the non-ARDSv and ARDSv tests expressed as Δ were performed. For pairwise comparisons, the Wilcoxon signed-rank test (when variables differed from normal distribution) or the paired *t*-test (when variables did not differ from normal distribution) was used. For the *t*-test, Cohen’s d was calculated as a measure of effect size. For the Wilcoxon signed-rank test, the effect size measure was *r*, calculated according to the formula [27]:

$$r=\frac{Z}{\sqrt{N}}$$

where *N* denotes the number of valid pairs and *Z* the test statistic calculated from the sum of ranks. In all analyses, *p* < 0.05 was considered statistically significant.

## 3. Results

**Figure 3:**
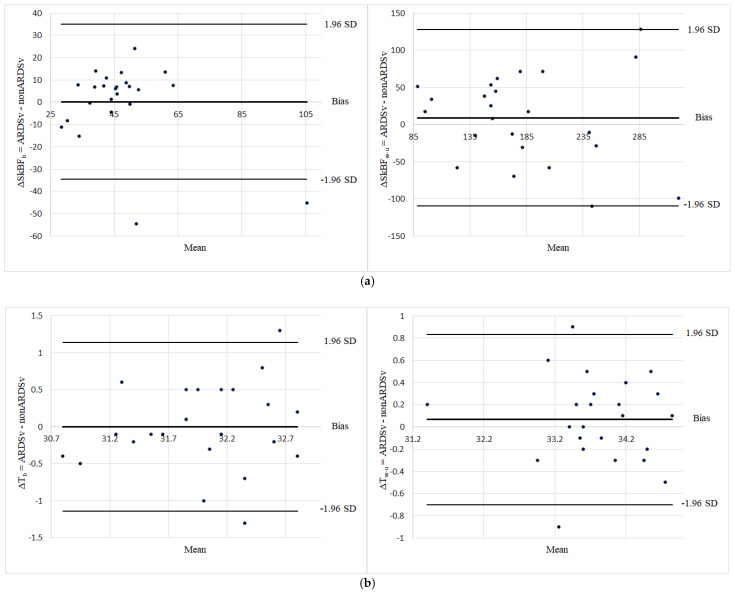

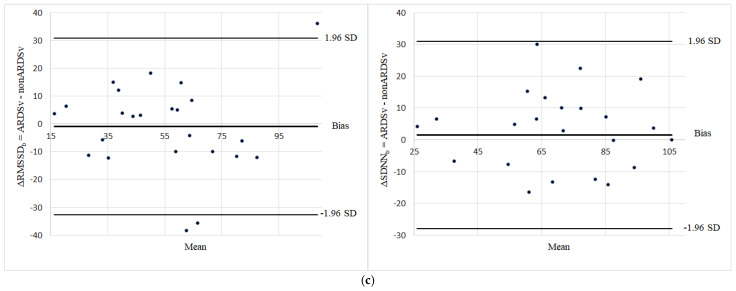
(**a**) Bland–Altman plots for skin blood flow. (**b**) Bland–Altman plots for skin surface temperature. (**c**) Bland–Altman plots for heart rate variability parameters.

Data on ICC(2.1), CV_intra/sub_, APD are presented in Table 1. Bland–Altman statistics are presented in Figure 3a–c. SEM and MDC_95_ were 12.25 and 33.95 for SkBF_b_, 42.09 and 116.66 for SkBF_w-u_, 0.40 and 1.11 for T_b_, 0.27 and 0.75 for T_w-u_, 11.14 and 30.89 for RMSSD_b_, and 10.39 and 28.79 for SDNN_b_. Moreover, SEM and MDC_95_ were 41.53 and 115.11 for ΔSkBF_0_, and 0.47 and 1.32 for ΔT_0_.

CV_inter/sub_ was 43.6%, 31.4%, 41.0%, and 35.0% for SkBF_b_ in the non-ARDSv and ARDSv tests and for SkBF_w-u_ in the non-ARDSv and ARDSv tests, respectively. In addition, CV_inter/sub_ was 1.9%, 2.2%, 2.4%, and 2.3% for T_b_ in the non-ARDSv and ARDSv tests and for T_w-u_ in the non-ARDSv and ARDSv tests, respectively. In the analysis of RMSSD_b_, CV_inter/sub_ was 43.8% and 44.4% for the non-ARDSv and ARDSv tests, respectively; in the analysis of SDNN_b_, CV_inter/sub_ was 31.8% and 29.7% for the non-ARDSv and ARDSv tests, respectively. In the analysis of differences, CV_inter_ was 55.4%, 47.1%, 36.0%, and 40.7% for ΔSkBF_0_ and ΔT_0_ in the non-ARDSv and ARDSv tests, respectively.

Basic statistics (means, standard deviations, and confidence intervals) for the above parameters are presented in Table 1.

The Shapiro–Wilk test revealed deviations from normal distribution for SkBF_2_ and SkBF_3_ in the non-ARDSv test, SkBF_1_, SkBF_2_, SkBF_3_, and SkBF_4_ in the ARDSv test, ΔSkBF_3_ and ΔSkBF_5_ in the non-ARDSv test, and ΔSkBF_3_ in the ARDSv test. The Friedman analysis of variance showed statistically significant effects of repeated measurements of SkBF in the non-ARDSv test (χ^2^ = 52.37; df = 4; *p* = 0.00; W = 0.55) and in the ARDSv test (χ^2^ = 64.1; df = 4; *p* = 0.00; W = 0.67). Statistically significant differences between repeated measurements are presented in Table 2.

All variables describing the temperature of the temple surface met the assumption of normal distribution. A two-factor analysis of variance showed an effect of repeated measurements for temple surface temperature (η^2^ = 0.65; df = 4; *p* = 0.00; F = 86.91). Statistically significant differences between repeated measurements are presented in Table 3.

It was demonstrated that only ΔSkBF 3 differed from a normal distribution in both the non-ARDSv and ARDSv tests. Using the paired *t*-test (or Wilcoxon test), no statistically significant differences were found in any comparisons between the non-ARDSv and ARDSv tests (Table 4).

In the non-ARDSv test, deviations from normal distribution were found for HFP_b_, HR_break_, RMSSD_break_, SDNN_break_, HFP_break_, and LFP_break_. In the ARDSv test, deviations from normal distribution were observed for HFP_b_, HR_break_, RMSSD_break_, SDNN_break_, HFP_break_, and LFP_break_. Table 5 shows statistically significant differences for the comparison between non-ARDSv and ARDSv.

In the non-ARDSv test, deviations from normal distribution were observed for HR_peak_. In the ARDSv test, deviations from normal distribution were found for HR_peak_, HRR1, HRR2, and HRR3. Analysis of variance revealed a repeated-measures effect for avRT (ƞ^2^ = 0.21; df = 1; *p* = 0.00; F = 11.97) and a mixed effect of repeated measures and test protocol for fRT (ƞ^2^ = 0.08; df = 1; *p* = 0.047; F = 4.15). Statistically significant results of the Bonferroni test are presented in Table 6. Analysis of covariance showed that avRT_b_ was a strong predictor of avRT_rec_ (ƞ^2^ = 0.61; df = 1; *p* = 0.00; F = 69.33) and the protocol used was a weak predictor of avRT_rec_ (ƞ^2^ = 0.07; df = 1; *p* = 0.08; F = 3.21).

## 4. Discussion

In the present study, to evaluate the skin blood flow between non-ARDSv and ARDSv conditions we used the results of skin surface temperature measurements on the temple because they achieved the required level of repeatability. However, the results of direct SkBF measurements did not meet the criteria adopted in this study. Therefore, the data from SkBF measurements should be interpreted with great caution and treated primarily as a premise for further studies using a larger number of measurement probes in a larger group of study participants. The presented study showed that differences between T_w-u_ and T_1_, as well as between T_w-u_ and T_2_, were similar in both the non-ARDSv and ARDSv procedures. These data indicate that ARDSv did not affect skin blood flow during the interval preceding the 3 min 110%Pmax test to an extent detectable by the applied measurement methods.

During intense exercise, there is a redistribution of blood flow to the pulmonary circulation and skeletal muscles [28,29]. However, this redistribution reduces blood flow to organs with lower metabolic demand during intense exercise [30]. In the initial phase of intense exercise, skin blood flow may decrease due to sympathetic activation, leading to cutaneous vasoconstriction [31]. These mechanisms are supported by the present findings, as SkBF and T during the 110%Pmax test differed significantly from both the end of the warm-up and the end of the pre-test interval in both protocols. At the same time, no statistically significant differences were obtained for T measurements between ARDSv and non-ARDSv conditions, which contradicts the hypothesis presented in the introduction regarding the body’s response to intense exercise.

Following short, high-intensity exercise, an increase in skin blood flow is typically observed, accompanied by a rise in skin surface temperature [7,32,33]. The present results indicate that only in the ARDSv protocol did the difference between recovery and exercise SkBF and T values reach statistical significance. This may suggest that post-exercise redistribution of blood flow to the skin may have been more pronounced in the ARDSv condition. The increase in skin blood flow results from the vasodilation of cutaneous vessels, involving reduced sympathetic vasoconstrictor activity and activation of sympathetic cholinergic pathways associated with thermoregulation during increased body temperature [34]. In the ARDSv protocol, a greater increase in body temperature may have occurred for several reasons: (a) a potentially lower SkBF during exercise preceded by breathing in ARDSv compared to non-ARDSv (a moderate effect, although not statistically significant, was described above); (b) higher oxygen uptake during exercise preceded by breathing in ARDSv compared to non-ARDSv [1], which could have promoted greater ATP production [35] and heat [36]; and (c) rebreathing of heated air from the preceding exhalation, similar to what occurs during diving or mask breathing [37]. These mechanisms could have promoted a higher thermal load on the body and influenced the regulation of cutaneous blood flow. However, the significance of these mechanisms requires confirmation in future studies that include measurements of core body temperature and airway temperature with the participation of a larger research group.

The available literature provides inconsistent findings regarding the effect of exercise on choice reaction time. Studies by Hebisz et al. [22] demonstrated that, in cyclists, choice reaction time improved several minutes after sprint interval training compared with resting values. Kashihara et al. [38] reported that choice reaction time improves after several minutes of exercise performed at lactate threshold intensity. However, Pavelka et al. [39] found that simple reaction time deteriorated in combat sports athletes following a Wingate test. These discrepancies suggest that post-exercise changes in reaction time may depend on the type of exercise preceding the measurement and/or sport-specific adaptations. The combined data presented in this study indicate that, following the applied testing procedure, reaction time measured in the fourth minute after completion of the 110%Pmax test improved compared with baseline values. Therefore, the present findings confirm earlier observations [22] that, in cyclists, choice reaction time improves following short, high-intensity exercise.

A separate analysis of the data showed that the protocol used differentiated the effects in terms of reaction time. It should be noted that the effect observed for fRT was statistically borderline (*p* = 0.047). Given the large number of comparisons performed in this study, this effect is subject to a significant risk of type I error. Furthermore, to clarify the effect for avRT, an additional analysis of covariance was performed, which showed that the covariate (baseline avRT measured before the exercise protocol) was a strong predictor of the effect (avRT measured after the 110%Pmax test). Simultaneously, the study protocol (non-ARDSv, ARDSv) was a weak predictor of the effect, which did not meet the criterion of statistical significance after taking into account the baseline performance. Therefore, the interpretation of the obtained results should be cautious at this stage. Confirmation of the existence of borderline statistical effects requires future studies conducted on larger groups using a modified protocol that includes the measurement of reaction time during breathing in ARDSv and during or immediately after intense exercise, preferably taking into account blood flow through the internal carotid artery, for example, indirectly using Doppler ultrasound.

Hypercapnia increases sympathetic activation while reducing parasympathetic activity [40]. For this reason, HR, RMSSD, SDNN, and HFP measured during the interval between the warm-up and high-intensity exercise were analyzed in the present study. Statistical analyses showed no significant differences between the non-ARDSv and ARDSv protocols in parameters reflecting vagal reactivation (RMSSD and HFP), with moderate effect sizes. These findings suggest that if ARDSv influenced vagal reactivation after the warm-up, the effect was subtle. An additional indicator of physiological stress and vagal reactivation is post-exercise heart rate recovery, particularly during the first few minutes after exercise cessation [41]. In the present study, post-exercise heart rate recovery was analyzed after completion of the 3 min 110%Pmax test. The results indicated no statistically significant differences between protocols for HRR. Therefore, breathing through ARDSv prior to intense exercise does not appear to constitute a major stressor, although it may slightly modify thermoregulatory responses to exercise and have a minor effect on reaction time.

As noted above, HRR during the first minutes of recovery is an indicator of vagal reactivation [41]. More precisely, HRR measured immediately after exercise primarily reflects the withdrawal of sympathetic activity [42]. Therefore, the moderate effect size observed for HRR1 may be due to slower withdrawal of sympathetic activity following intense exercise in the ARDSv protocol compared with the non-ARDSv protocol. Furthermore, the present analysis revealed differences in HR_break_, SDNN_break_, and LFP_break_ between the non-ARDSv and ARDSv protocols. These findings indicate that ARDSv affects overall heart rate variability and components associated with slower oscillations of the autonomic nervous system, even if short-term parasympathetic indices (RMSSD and HFP) remain stable. These parameters reflect distinct aspects of autonomic balance and partially explain sympathetic modulation [43,44], which is a known effect of elevated pCO_2_ [45]. Additionally, they may partly reflect increased baroreflex activation [46]. Although these changes were too small to significantly affect stress levels (as reflected by RMSSD and HFP), they may have contributed to a faster cardiorespiratory response. Consequently, they may help explain the previously reported increase in oxygen uptake during exercise following ARDSv breathing [1]. A transient increase in sympathetic activation may be a likely mechanism explaining the previously described ARDSv effects on skin blood flow and reaction time. Increased sympathetic activity may influence vascular tone in both the skin circulation and the central nervous system, potentially contributing to blood flow redistribution. For example, Charkoudian [47] described the sympathetic adrenergic vasoconstrictor system and the active vasodilator system involved in the regulation of skin blood flow. Ter Laan et al. [48] described sympathetic mechanisms that protect the brain from excessive increases in cerebral blood flow. In the context of the present study, sympathetic mechanisms could therefore indirectly influence SkBF and RT responses.

### Limitations

The statistical power analysis assumed a priori the presence of large statistical effects. The effect sizes obtained in some of the SkBF comparisons (Cohen’s d ≈ 0.3) indicate that the actual effects were smaller than anticipated at the study planning stage. This means that the assumptions made may have led to an underestimation of the required sample size. Therefore, future studies should include larger study groups to enable the detection of small and moderate statistical effects. Furthermore, the η^2^ values observed in selected comparisons differed from the assumptions made during the planning phase of the present study. This may indicate an inaccuracy in the required sample size estimate resulting from the adopted effect size assumptions. The effect size for statistical power analysis was estimated based on conventional η^2^ interpretation thresholds (small/moderate/large) used in the methodological literature [49,50]. This procedure was adopted due to the lack of previous empirical data on the analyzed physiological stimuli (ARDSv or similar ones, e.g., hypercapnia) in comparable exercise models. The obtained results may serve as a reference point for more precise selection of a priori assumptions in future studies in this area.

Furthermore, a limitation of the present study is that the choice reaction time measurements were only performed before the warm-up and after the 110% Pmax test. Before this study began, an additional reaction time measurement between the warm-up and the 110% Pmax test was considered. However, including such a measurement could have confounded other concurrent measurements, particularly heart rate variability (HRV), which required maintenance conditions without additional cognitive testing. Therefore, a methodological compromise was adopted by limiting the number of reaction time measurements to allow for the simultaneous assessment of several physiological and cognitive parameters within a single research protocol.

In the present study, participants were instructed to maintain their dietary and hydration habits throughout this study. However, detailed data regarding dietary intake and hydration were not formally monitored or recorded, which is certainly a limitation of this study and should be included in future studies.

## 5. Conclusions

Higher heart rate and changes in selected HRV parameters during the period between warm-up and the 3 min test suggest increased physiological strain associated with the ARDSv procedure. Furthermore, weak and inconclusive effects were observed for skin blood flow and choice reaction time. Due to the limitations of the present study, these findings require confirmation in larger cohorts and with the use of measurement methods providing greater repeatability and reliability.

## Figures and Tables

**Table 1:** Summary of parameters used to assess measurement repeatability between the non-ARDSv and ARDSv tests.

Variables	Non-ARDSv	ARDSv	CVintra	ICC(2.1)	APD	*p*
	$\bar{x}$ ± SD	$\bar{x}$ ± SD	%		%	
**SkBF_b_ [PU]**	47.1 ± 20.1	47.3 ± 14.4	26	0.5	−1.59 ± 33.0	0.22
**T_b_ [°C]**	32.0 ± 0.6	32.0 ± 0.7	1.3	0.59	0.01 ± 1.8	1.00
**SkBFw-u [PU]**	178.4 ± 71.3	187.4 ± 64.0	23.1	0.61	−7.37 ± 31.1	0.47
**Tw-u [** **°C** **]**	33.8 ± 0.8	33.8 ± 0.7	0.8	0.85	−0.20 ± 1.16	0.41
**ΔSkBF_0_ [PU]**	131.3 ± 72.8	140.2 ± 66.0	30.7	0.64	−10.36 ± 46.31	0.47
**Δ** **T_0_ [** **°C** **]**	1.78 ± 0.64	1.84 ± 0.75	26.3	0.53	−2.00 ± 69.49	0.64
**RMSSD_b_ [ms]**	55.2 ± 23.6	54.2 ± 23.6	20.5	0.77	0.32 ± 28.24	0.78
**SDNN [ms]**	70.3 ± 22.4	71.7 ± 21.3	14.7	0.77	−2.93 ± 21.31	0.63

SkBF—skin blood flow; T—body surface temperature; RMSSD—root mean square of successive differences between RR intervals; SDNN—standard deviation of the mean distance between intervals RR; Δ—the difference between the measurement taken during the warm-up and the measurement taken before the start of the warm-up; _b_—measurement before the start of the warm-up; w-u—measurement during the warm-up; x—mean value; ARDSv—measurement in the protocol with ARDSv; non-ARDSv—measurement in the protocol without ARDSv; SD—standard deviation; APD—absolute percentage difference; *p*—level of statistical significance for differences between measurements.

**Table 2:** Skin blood flow in cyclists in the non-ARDSv and ARDSv tests.

Variables	Non-ARDSv		ARDSv	
	$\bar{x}$ ± SD	LCI—UCI	$\bar{x}$ ± SD	LCI—UCI
**SkBF_w-u_ [PU]**	178.4 ± 71.3	148.3–208.5	187.4 ± 64.0	160.4–214.5
**SkBF_1_ [PU]**	147.0 ± 66.3 * SkBF_w-u_	119.0–174.9	161.2 ± 65.2 * SkBF_w-u_	133.7–188.7
**SkBF_2_ [PU]**	114.5 ± 68.1 * SkBF_w-u_	85.7–143.3	112.8 ± 56.1 * SkBF_w-u_	89.1–136.5
**SkBF_3_ [PU]**	108.3 ± 47.9 * SkBF_w-u_	88.1–128.6	103.2 ± 39.9 * SkBF_w-u_	86.3–120.0
**SkBF_4_ [PU]**	121.5 ± 45.7	102.2–140.8	125.9 ± 40.2 * SkBF_3_	108.9–142.9

SkBF—skin blood flow; _w-u_—measurement during the warm-up; _1,2,3,4_—measurement respectively: in the first half of the warm-up, in the second half of the warm-up, during the test at 110%Pmax, during recovery after the test at 110%Pmax; x—mean value; ARDSv—measurement in the protocol with ARDSv; non-ARDSv—measurement in the protocol without ARDSv; SD—standard deviation; LCI—lower confidence interval; UCI—upper confidence interval; *—*p* < 0.05.

**Table 3:** Temple surface temperature in cyclists in the non-ARDSv and ARDSv tests.

Variables	Non-ARDSv		ARDSv	
	$\bar{x}$ ± SD	LCI—UCI	$\bar{x}$ ± SD	LCI—UCI
**T_w-u_ [** **°C** **]**	33.8 ± 0.6	33.4–34.1	33.8 ± 0.7	33.5–34.1
**T_1_ [** **°C** **]**	33.5 ± 0.8 * T_w-u_	33.2–33.8	33.6 ± 0.8 * T_w-u_	33.3–34.0
**T_2_ [** **°C** **]**	32.6 ± 0.8 * T_w-u_	32.3–33.0	32.8 ± 0.9 * T_w-u_	32.4–33.1
**T_3_ [** **°C** **]**	32.1 ± 1.1 * T_w-u_, T_2_	31.7–32.6	32.3 ± 1.0 * T_w-u_, T_2_	31.8–32.7
**T_4_ [** **°C** **]**	32.5 ± 1.1	102.2–140.8	32.8 ± 1.0 * T_3_	32.4–33.2

T—body surface temperature measured at the temple; w-u—measurement during the warm-up; _1,2,3,4_—measurement respectively: in the first half of the warm-up, in the second half of the warm-up, during the test at 110%Pmax, during recovery after the test at 110%Pmax; x—mean value; ARDSv—measurement in the protocol with ARDSv; non-ARDSv—measurement in the protocol without ARDSv; SD—standard deviation; LCI—lower confidence interval; UCI—upper confidence interval; *—*p* < 0.05.

**Table 4:** Changes in skin blood flow in cyclists during the non-ARDSv and ARDSv tests.

Variables	Non-ARDSv		ARDSv		Effect Size
	$\bar{x}$ ± SD	LCI—UCI	$\bar{x}$ ± SD	LCI—UCI	
**ΔSkBF_1_ [PU]**	−31.4 ± 29.3	−43.8–−19.1	−26.2 ± 21.5	−35.3–−17.2	d = 0.20
**ΔSkBF_2_ [PU]**	−63.9 ± 56.3	−87.6–−40.1	−74.7 ± 56.6	−98.6–−50.8	d = 0.19
**ΔSkBF_3_ [PU]**	−6.2 ± 29.0	−18.5–6.1	−9.6 ± 42.4	−27.5–8.3	d = 0.19
**ΔSkBF_4_ [PU]**	−70.1 ± 50.3	−91.3–−48.8	−84.3 ± 40.2	−104.6–−63.9	d = 0.31
**ΔSkBF_5_ [PU]**	13.2 ± 34.6	−1.4–27.8	22.7 ± 31.9	9.2–36.1	d = 0.29

ΔSkBF—change in skin blood flow; ΔSkBF_1_ = SkBF_1_ − SkBF_w-u_; ΔSkBF_2_ = SkBF_2_ − SkBF_w-u_; ΔSkBF_3_ = SkBF_3_ − SkBF_2_; ΔSkBF_4_ = SkBF_3_ − SkBF_w-u_; ΔSkBF_5_ =SkBF_4_ − SkBF_3_; ARDSv—measurement in the protocol with ARDSv applied; non-ARDSv—measurement in the protocol without ARDSv; SD—standard deviation; LCI—lower confidence interval; UCI—upper confidence interval; d—Cohen’s d, r—effect size for Wilcoxon paired test.

**Table 5:** Heart rate and heart rate variability in cyclists under baseline conditions and during the interval preceding the 3 min non-ARDSv and ARDSv tests.

Variables	Non-ARDSv		ARDSv		Effect Size
	$\bar{x}$ ± SD	LCI—UCI	$\bar{x}$ ± SD	LCI—UCI	
**HR_b_ [bpm]**	80.1 ± 15.9	73.3–86.8	79.0 ± 14.7	72.8–85.2	d = 0.07
**RMSSD_b_ [ms]**	55.2 ± 23.6	45.2–65.1	54.2 ± 23.6	44.3–64.2	d = 0.04
**SDNN_b_ [ms]**	70.3 ± 22.4	60.8–79.7	71.7 ± 21.3	62.7–80.8	d = 0.06
**HFP_b_ [ms^2^]**	1497.7 ± 1187.2	996.3–1999.0	1532.3 ± 1438.2	925.0–2139.6	r = 0.05
**LFP_b_ [ms^2^]**	3162.6 ± 2005.9	2315.6–4009.6	3443.4 ± 1730.6	2712.6–4174.2	d = 0.15
**HR_break_ [bpm]**	114.1 ± 15.0	107.8–120.5	117.7 ± 15.7 *	111.1–124.4	r = 0.60
**RMSSD_break_ [ms]**	17.6 ± 17.8	10.1–25.1	15.1 ± 16.7	8.0–22.1	r = 0.33
**SDNN_break_ [ms]**	25.4 ± 14.2	19.5–31.4	20.8 ± 13.6 *	15.1–26.6	r = 0.73
**HFP_break_ [ms^2^]**	178.4 ± 342.3	33.9–322.9	95.0 ± 159.1	27.8–162.2	r = 0.33
**LFP_break_ [ms^2^]**	512.7 ± 657.7	235.0–790.5	417.0 ± 839.3 *	62.6–771.4	r = 0.54

HR—heart rate; RMSSD—root mean square of successive differences between RR intervals; SDNN—standard deviation of RR intervals; HFP—high-frequency power; LFP—low-frequency power; _b_—measurement taken before the warm-up; _break_—measurement taken during the interval between the warm-up and the 110%Pmax test; ARDSv—measurement in the protocol with ARDSv applied; non-ARDSv—measurement in the protocol without ARDSv; SD—standard deviation; LCI—lower confidence interval; UCI—upper confidence interval; d—Cohen’s d, r—effect size for Wilcoxon paired test. *—*p* < 0.05 vs. non-ARDSv.

**Table 6:** Recovery heart rate and choice reaction time in cyclists after the 3 min non-ARDSv and ARDSv tests.

Variables	Non-ARDSv		ARDSv		Effect Size
	$\bar{x}$ ± SD	LCI—UCI	$\bar{x}$ ± SD	LCI—UCI	
**HR_peak_ [bpm]**	193.2 ± 12.5	187.9–198.5	194.5 ± 11.4	189.6–199.3	r = 0.29
**HRR1 [bpm]**	156.4 ± 19.6	148.1–164.7	158.9 ± 15.5	152.4–165.5	r = 0.29
**HRR2 [bpm]**	133.2 ± 16.9	126.1–140.4	134.1 ± 15.5	127.6–140.7	r = 0.02
**HRR3 [bpm]**	123.9 ± 13.8	118.1–129.7	123.2 ± 12.8	117.8–128.6	r = 0.17
**ΔHRR1 [bpm]**	36.8 ± 13.1	31.3–42.4	35.5 ± 12.0	30.5–40.6	d = 0.10
**ΔHRR2 [bpm]**	60.0 ± 10.1	55.7–64.2	60.3 ± 13.5	54.6–66.0	d = 0.03
**ΔHRR3 [bpm]**	69.3 ± 8.7	65.6–73.0	71.3 ± 12.2	66.1–76.4	d = 0.19
**avRT_b_ [s]**	0.38 ± 0.06	0.36–0.40	0.37 ± 0.04	0.35–0.39	---
**avRT_rec_ [s]**	0.35 ± 0.05 *	0.33–0.37	0.36 ± 0.05	0.34–0.38	---
**fRT_b_ [s]**	0.27 ± 0.04	0.25–0.29	0.26 ± 0.04	0.24–0.27	---
**fRT_rec_ [s]**	0.26 ± 0.05	0.24–0.28	0.26 ± 0.04	0.25–0.28	---

HR_peak_—peak heart rate during the 110%Pmax test; HRR—recovery heart rate at 1, 2, or 3 min after completion of the 110%Pmax test; ΔHRR—difference between HR peak and HRR measured at 1, 2, or 3 min of recovery; avRT—mean reaction time; fRT—fastest reaction time recorded in the test; _b_—baseline reaction time measurement; _rec_—recovery reaction time measurement; ARDSv—measurement in the protocol with ARDSv applied; non-ARDSv—measurement in the protocol without ARDSv; SD—standard deviation; LCI—lower confidence interval; UCI—upper confidence interval; d—Cohen’s d; r—effect size for Wilcoxon paired test; *—*p* < 0.05 vs. baseline value.

## Data Availability

Data will be provided on request.

## Abbreviations

The following abbreviations are used in this manuscript:

VO_2_max	Maximal oxygen uptake
Pmax	Maximal power output in the progressive test
VE	Minute ventilation
VCO_2_	Carbon dioxide output
RER	Respiratory exchange ratio
PETO_2_	End-tidal partial pressure of oxygen
PETCO_2_	End-tidal partial pressure of carbon dioxide
VT1	First ventilatory threshold
VT2	Second ventilatory threshold
SkBF	Skin blood flow
ΔSkBF	Change in skin blood flow
T	Body surface temperature
HR	Heart rate
HR peak	Peak heart rate during the 110%Pmax test
HRR	Recovery heart rate at 1, 2, or 3 min after completion of the 110%Pmax test
ΔHRR	Difference between HR peak and HRR measured at 1, 2, or 3 min of recovery
RMSSD	Root mean square of successive differences between RR intervals
SDNN	Standard deviation of RR intervals
HFP	High-frequency power
LFP	Low-frequency power
non-ARDSv	Measurement in the protocol without ARDSv
ARDSv	Measurement in the protocol with ARDSv
avRT	Mean reaction time
fRT	Fastest reaction time recorded in the test
